# Experimental study on the permeability of crushed coal medium based on the Ergun equation

**DOI:** 10.1038/s41598-021-02524-4

**Published:** 2021-11-29

**Authors:** Mingkun Pang, Tianjun Zhang, Yukai Meng, Zhiqiang Ling

**Affiliations:** 1 Key Laboratory of Western Mine Exploitation and Hazard Prevention of the Ministry of Education, Xi’an, People’s Republic of China; 2 College of Safety Science and Engineering, Xi’an University of Science and Technology, 58, Yanta Mid. Rd., Xi’an, 710054 Shaanxi People’s Republic of China

**Keywords:** Hydrology, Natural hazards, Solid Earth sciences, Hydrology

## Abstract

Accurate determination of the permeability of crushed coal medium is the basis for the study of their permeability characteristics. To investigate the permeability characteristics of this special porous medium composed of crushed coal particles, the permeability parameters of crushed coal specimens of different initial porosities were measured by designing a lateral-limit compression seepage test system. Parameters were determined separately for specimens of different initial porosities. (1) the Reynolds number distribution region characterising the seepage state was determined and obtained. Specimens with initial porosity distribution between 0.02 and 0.08, and seepage Reynolds number distribution in the low-permeability zone, under Darcy flow; (2) the intrinsic permeability of the crushed coal medium was obtained by using the Ergun equation. The complex inverse proportional relationship between the drag coefficient and Reynolds number was derived; (3) Through the determination of the permeability of the crushed coal medium, the mean value of *βK* value was obtained to be about 45.7, and the analysis of the permeability of porous medium can determine its critical permeability. The relationship between the Forchheimer number *F*o and critical Reynolds number was measured. The results indicate that it conforms to a linear distribution. In-depth analysis of these two parameters can be used to explore the flow transition process between laminar, transition, and turbulent flow. This study provides insight into the permeability characteristics of the media in fractured coal bodies.

## Introduction

Percolation in crushed coal grains has been a matter of interest and has a very long history of research^1,2^. However, this porous medium composed of crushed coal grains demonstrates more complex permeability characteristics. From one viewpoint, its structure is influenced by factors such as pore size, pore shape, and porosity^3–5^. Therefore, even the most basic and commonly used model equations for the parametric characteristics of this particular medium remain under investigation. Ergun^6^ (in 1952) published his original relational equation based on porosity and grain size to describe porous media by studying the physical model of air flow in porous media. Equation (1) is the classical Ergun equation.

1 $$- \frac{dP}{{dx}} = \alpha \frac{{\left( {1 - \varepsilon } \right)^{2} \mu }}{{\varepsilon^{3} D_{p}^{2} }}v + \beta \frac{{\left( {1 - \varepsilon } \right)\rho }}{{\varepsilon^{3} D_{p} }}v_{{}}^{2}$$

where $\alpha$ represents the coefficient of viscous resistance term, $\beta$ is the coefficient of formal resistance term, $\mu$ is the gas viscosity, *x* is displacement, $\varepsilon$ denotes porosity, and $D_{p}$ refers to grain diameter. Accurate determination of the permeability of porous media consisting of crushed coal grains is seen as a difficult task^7–9^, which is important for studying the analysis of the permeability characteristics of crushed coal grains and porous coal media in mines.

The permeation mechanism of porous media composed of crushed coal grains and the applicability of their calculation methods have been widely studied^10,11^. The traditional Forchheimer equation or Ergun equation mainly includes the effects of viscosity and inertia, however, the composition and structure of arbitrary porous media cannot be characterized by porosity variations alone^12–14^. Changes in grain distribution or grain shape and size can alter the curvature and connectivity of the pore space, thus changing the overall permeability properties of the medium^15–17^. Dukhan^18^ evaluated the validity of the Ergun-type relationship to confirm the correlation between permeability and porosity, thus providing easy-to-use, quantitative, pore-permeability equations. Based on the Ergun equation, Du Plessis^19^ theoretically derived the momentum transport equation for fully developed flow in porous media. Dukhan and Mohamed^20^ investigated the effects of wall surface and size on permeability and shape drag coefficient by laboratory experiments. Cheng^3^ presented a capillary-type model on a modified basis to estimate the effect of pressure drop on the wall surface of a packed bed using the Ergun equation. Mehta and Hawley^21^ used a modified Ergun equation to account for wall effects on friction. It is worth noting that although the modified Ergun equation predicts the experimental results well^22,23^, this limits to some extent the form of fixed expressions it uses and introduces the question of whether its parameters can be calculated accurately in all cases.

So far, although much research has been conducted on the crushed coal medium in terms of experiments: there are many fitting formulae used in conjunction with engineering applications, but the accurate determination of the permeability parameters of this medium has not been unified in terms of its form of calculation. Different methods for the experimental determination of its parameters give different meanings. More rigorous tests were warranted to determine its parameters, and explore the dependence between such parameters. Therefore. a series of parameters of the crushed coal medium, such as critical Reynolds number, friction factor, and permeability, were determined by introducing the calculation method of effective porosity using the modified Ergun equation with a porous medium composed of crushed coal grains as the test object. In addition, the magnitude range of *βK* values, and the relationship between correlation Fo and Re were also studied. In turn, the basic permeability characteristics of the crushed coal grain medium were characterised by the variations in parameters.

## Theoretical background

### Classical definition of effective porosity

The crushed coal medium is a special porous medium composed of a skeleton made of crushed coal grains, and pores. It is difficult to be characterized by precise mathematical methods, and the fluid flow path is tortuous and complex, the magnitude and direction of the mass flow velocity changes frequently, and the frictional resistance is significant.

**Figure 1:**
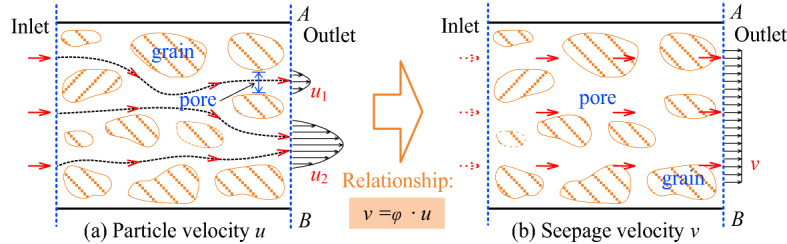
Basic definition of seepage velocity.

By introducing the concept of unitary volume of porous media and defining the porosity under the representative elementary volume (REV), it is possible to equate the porous medium as a continuum medium, so that the porous medium demonstrates porosity everywhere, where $\phi$ is the ratio of the pore volume to the total volume, called the effective porosity: the mass flow rate and seepage flow rate satisfy the relationship, as shown in Fig. 1.

### Classical expressions for the Ergun equation

Fluid percolation at higher velocities usually exhibits non-Darcian flow due to inertial forces and turbulent effects, and non-Darcy flow caused by high flow velocities becomes increasingly important^24,25^. For the infiltration of porous media with high velocity flow, the classical form of the Forchheimer equation was given by Forchheimer in 1901^26^, as per Eq. (2).

2 $$\frac{\Delta P}{L} = \frac{\mu }{K}v + \beta \rho v^{2}$$

where *P* denotes permeation pressure; *L* is seepage path length; *μ* is dynamic viscosity; *K* is permeability (Forchheimer type); *v* represents permeation velocity (Forchheimer type); *β* denotes non-Darcy coefficient; and *ρ* is fluid density.

The non-Darcian factors in the Forchheimer equation have been broadly studied^27–29^. The Ergun equation, was the most extensively studied, which was summarised by Ergun based on the theory of pipe flow, and the definition of the mean hydraulic radius. Ergun gave a convenient expression for the equation as calculated from 640 sets of experimental data.

3 $$\frac{\Delta P}{L} = \frac{{150\mu \left( {1 - \phi } \right)^{2} v_{0} }}{{D_{p}^{2} \phi^{3} }} + \frac{{1.75\rho \left( {1 - \phi } \right)v_{0}^{2} }}{{D_{p} \phi^{3} }}$$

where $\phi$ denotes porosity; $v_{0}$ represents permeability velocity (Ergun type); and $D_{p}$ is equivalent particle size. The first term on the right-hand side of the equal sign is the Blake–Kozeny equation, which is only applicable to the laminar flow zone, for which $\left( {{{D_{p} \rho v_{s} } \mathord{\left/ {\vphantom {{D_{p} \rho v_{s} } \mu }} \right. \kern-\nulldelimiterspace} \mu }} \right)\left( {1 - \phi } \right)^{ - 1} < 10$. The second term to the right of the equals sign is the Burke-Plummer equation, which is applicable to turbulent flow, the region is $\left( {{{D_{p} \rho v_{s} } \mathord{\left/ {\vphantom {{D_{p} \rho v_{s} } \mu }} \right. \kern-\nulldelimiterspace} \mu }} \right)\left( {1 - \phi } \right)^{ - 1} > 100$. To derive the formula, we also made the following assumptions:

① The flow is stable and fully developed;
② The porous medium is isotropic and homogeneous, so its property parameters are constant;
③ Flow occurs only in the horizontal direction;
④ The flow in the porous medium remains isothermal.

### Permeability expression of the Ergun equation

Usually, the calculation of parameters for Forchheimer-type non-Darcy flow consists of three main steps. First, a curve of the infiltration pressure gradient versus the flow velocity is plotted. Second, the curve is fitted as a function of the form *y* = a*x* + b*x*^2^, where a = *μ*/*k* and b = *ρβ*. Finally, the values of *μ* and *ρ* are introduced and the values of *k* and *β* are back-calculated. The form of the calculation of the viscous resistance coefficient under the definition of the Ergun equation, and the form of the calculation of the inertial resistance coefficient are derived as follows:

Viscous resistance coefficient:

4 $$A_{Ergun} = 150 \cdot \frac{{\left( {1 - \phi } \right)^{2} }}{{\phi^{3} }} \cdot \frac{\mu }{{D_{p}^{2} }}$$

Inertial resistance coefficient:

5 $$B_{Ergun} = 1.75 \cdot \frac{{\left( {1 - \phi } \right)}}{{\phi^{3} }} \cdot \frac{\rho }{{D_{p} }}$$

By comparing the Forchheimer equation (Eq. 2) and the Ergun equation (Eq. 3), expressions for the variations in permeability *K* and *β* coefficient with porosity and grain size were derived (Eqs. 6 and 7), respectively. This is very similar to the Kozeny–Carman permeability form given by Bear^30^, except for the constants. The coefficient *β* is essential in the calculation of Fo. Several correlations are proposed for *β*, which can be written in a generalised form, and Lopez et al.^31^ published a review of such correlations. the expression for the permeability *K* under the definition of the Ergun equation (Eq. 6), and the expression for the non-Darcy factor *β* (Eq. 7), are shown below.

Permeability *K*:

6 $$K_{Ergun} = \frac{{D_{p}^{2} \phi^{3} }}{{150\left( {1 - \phi } \right)^{2} }}$$

Non-Darcy factor *β*:

7 $$\beta_{Ergun} = 1.75\frac{{\left( {1 - \phi } \right)}}{{D_{p} \phi^{3} }}$$

## Experiments

Using the physical test method to determine the permeability of crushed coal grains, the permeability characteristics of the crushed coal medium can be studied, and more importantly, the parameters in the non-linear percolation equation can be validated. The permeability *K* and parameter *β* under the definition of the Ergun equation are verified.

### Materials

#### Research background

Shaanxi Heyang Coal Mine is located between Chengcheng and Heyang Counties, north-east of Xi’an city, Shaanxi Province, China: the well field is located in the eastern part of WeiBei coal field, the production capacity is 1.2 Mt/a, the commercial coal is the national scarce high quality power coal. 5# coal is the main mineable coal seam, its original moisture is 0.76%, the average thickness is 4.5 m, the main roof is 10.0-m thick K4 medium grain sandstone, the immediate roof is dominated by 1.15-m thick sandy mudstone. The floor is 8.6-m thick K3 quartz sandstone. It is a typical “three-soft” coal seam, with an unstable roof, loose coal seam, a small solidity coefficient, being easy to flake, with a soft bottom plate, and a low compressive strength.

#### Material preparation

In this test, the coal blocks were taken from Heyang coal mine, and the coal crystals of different grain sizes (including four grain sizes 0.5 mm, 1.0 mm, 1.5 mm, and 2.0 mm) were determined through crushing and sorting. Considering the accommodating volume of the test cylinder, and the volume compression ratio of the loaded specimens, each group of specimens relied on a filling height of about 100 mm and a total mass of about 500 g. Talbot grading theory was then used to calculate the mass ratio of each grain size range, and the number of the blended crushed coal samples was calculated according to the Talbot power index *n*, Sample 1, Sample 2, …, etc. (Table 1).

**Table 1:** The ratio of particle sizes in the sample.

Grain size/mm	Different values of the Talbot index are taken for *n*								
	0.1	0.2	0.3	0.4	0.5	0.6	0.7	0.8	0.9
0.5	435.3	378.9	329.9	287.2	250.0	217.6	189.5	164.9	143.6
1.0	31.2	56.3	76.2	91.8	103.6	112.2	118.3	122.2	124.4
1.5	19.3	36.8	52.5	66.7	79.5	90.9	101.0	110.0	118.0
2.0	14.2	28.0	41.3	54.3	67.0	79.3	91.2	102.8	114.1

### Methodologies

#### Experiment design

The test process is divided into the following steps: (1) crushing and sieving coal specimens with different grain sizes; (2) preparing specimens containing different initial gradation structures according to the Talbot formula; (3) loading the specimens into the infiltration cylinder in the order of coded specimens for pre-compaction; (4) conducting infiltration tests according to the preset axial pressure gradient, the infiltration pressure gradient, and recording the data. In the experiments, four gradients of axial displacement control 5, 10, 15, and 20 mm were adopted to load the coal specimens with different grading structures for progressive loading percolation tests. Each axial loading test was conducted at seepage pressures of 0.5, 1.0, 1.5, 2.0, and 2.5 MPa, respectively, and the axial loading *F*, axial displacement Δ*h*, and loading time *t* were recorded on the computer system.

#### Experimental equipment

**Figure 2:**
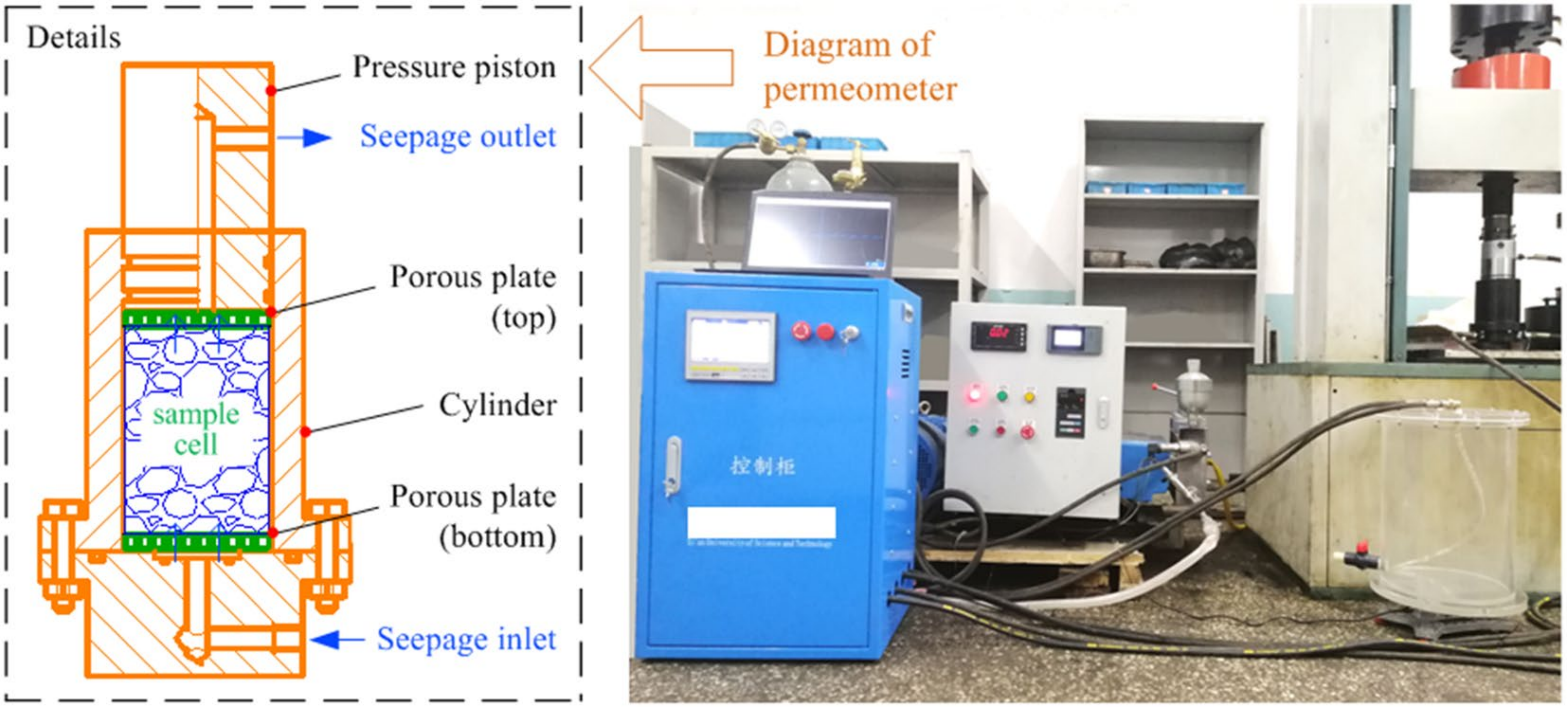
Sample installation diagram.

The lateral limit compression percolation test system used is a part of our homemade apparatus, the main structure of which includes: an axial pressure control module, permeation pressure control module, and a data acquisition module; the system can deliver axial pressure within the range from 0 to 200 kN, displacement control between 0.05 and 500 mm/min, a permeation pressure range of up to 16 MPa, etc. The permeate fluid medium can be water (*μ* = 1.01 × 10^–3^ Pa s), oil (*μ* = 1.96 × 10^–2^ Pa s), helium (*μ* = 1.89 × 10^–5^ Pa s). In this experiment, water was chosen as the seepage liquid. A schematic representation of the specimen installation is shown in Fig. 2.

## Results and discussion

By designing and conducting physical tests, we have determined the parameters of crushed coal grains during permeation. To analyse their permeation characteristics, a series of parameters are discussed including: calculation of critical Reynolds number, analysis of friction factor, analysis of the magnitude of the *βK* values, and correlation of Fo with Re.

### Distribution of permeability Reynolds number of crushed coal body

According to its definition in fluid mechanics, the Reynolds number Re can be used to determine whether the seepage of fluid in porous media obeys Darcy’s law of seepage: it is an important indicator used to determine the state of motion of viscous fluids.

When Re is greater than the lower critical Reynolds number Re_cr_, the fluid flow is laminar; when Re is greater than the upper critical Reynolds number Re_cr_, the fluid flow is turbulent; when Re is greater than the lower Reynolds number Re_cr_ and lower than the upper Reynolds number Re'_cr_, laminar flow and turbulent coexist, the flow is extremely unstable, if there is an external disturbance, the laminar flow may instantly change to a turbulent state. The critical Reynolds number is calculated as in Eq. (8).

8 $$Re_{cr} = \frac{{\rho v_{cr} d}}{\mu } = \frac{{v_{cr} d}}{\upsilon }$$

where *ρ* is the density of the fluid; *v*_cr_ is the average velocity of fluid penetration; *μ* denotes the dynamic viscosity of the fluid; *υ* represents the kinematic viscosity of the fluid; and *d* is the equivalent diameter of the flow channel (the diameter of the coal sample in this study).

**Figure 3:**
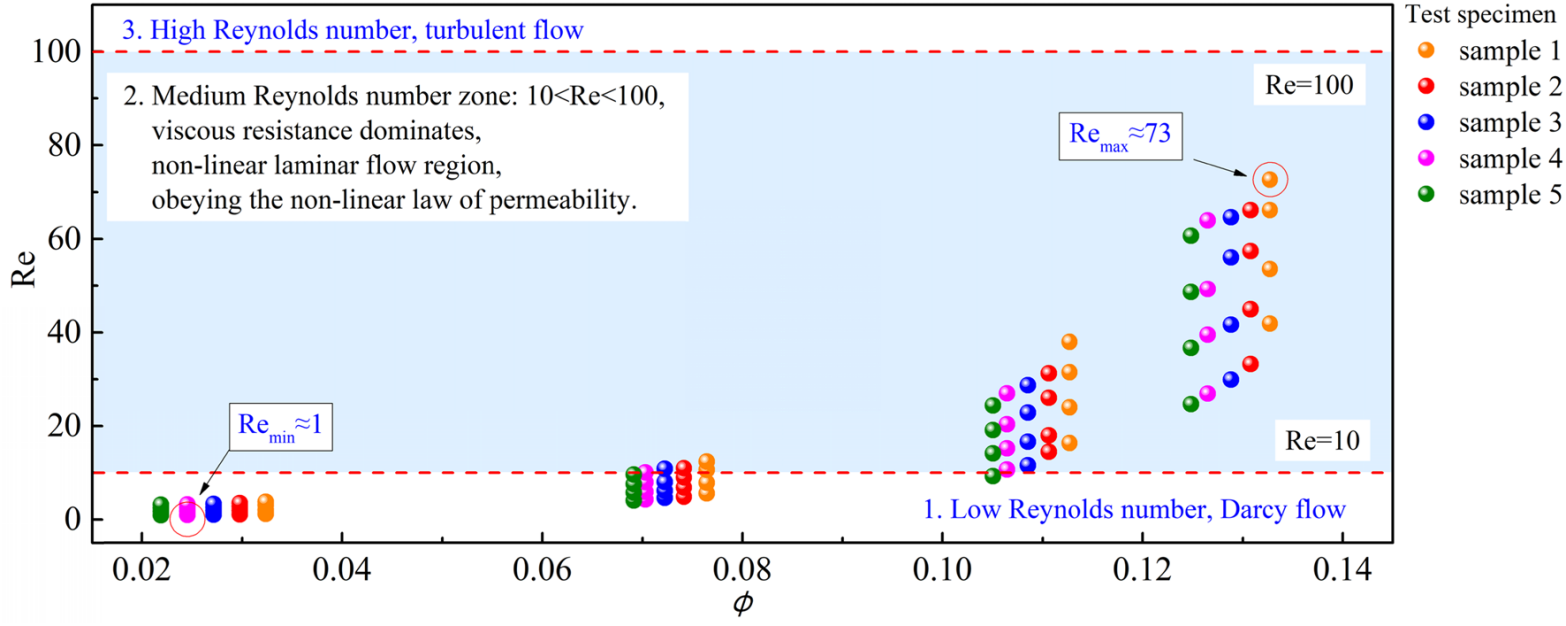
Characteristics of Reynolds number distribution of each group of specimens.

A large number of studies have shown that the flow of gas in crushed coal bodies can be divided into three zones^28^: (1) Low Reynolds number zone: Re < 10, wherein viscous drag dominates, classifying it as the linear laminar flow region, wherein the flow follows Darcy’s law; (2) medium Reynolds number zone: 10 < Re < 100, wherein viscous resistance dominates, classifying it as a non-linear laminar flow region, wherein non-linear permeability behaviour predominates; (3) A high Reynolds number zone: Re > 100, wherein turbulent flow occurs, inertial forces dominate, and the flow resistance is proportional to the square of the flow velocity. The Re number is shown in Fig. 3.

By adjusting the permeation pressure and obtaining the percolation volume *Q* for different groups of specimens at different pressures, the flow rate *v* can be calculated, and the corresponding Reynolds number at different flow rates is deduced. This can be used as a criterion to judge the behaviour of non-Darcy flow. In the test, the Reynolds number distribution of all groups of specimens ranged from 1 to 73 and the Reynolds number distribution could be divided into three intervals: a high Reynolds number interval, a medium Reynolds number interval, and a low Reynolds number interval. The specimens have porosity in the range of 0.02–0.08, whose permeability Reynolds numbers were in the low-permeability region, which showed that 45% of the specimens conformed to Darcy flow in the permeability process. For the specimens with porosity in the range of 0.10–0.14, the dynamic process is more complex, belonging to the conforming flow state. The distribution area of the seepage Reynolds number is divided, and this work can reflect the seepage state of different initial pore coal samples at different permeability pressures.

### Analysis of seepage drag coefficient of crushed coal body

At low flow rates, where the pressure drop is proportional to the flow rate and fluid viscosity, the intrinsic permeability is determined according to Darcy’s law. To calculate the permeability of porous media from the pressure gradient and flow rate under Darcian conditions, Chor and Li^32^ considered that the linear deviation due to inertial losses can be used as a function of porosity to estimate the Reynolds number by use of Eq. (9).

9 $${\text{Re}}_{p} = \frac{{D_{p} \rho v_{0} }}{{\mu \left( {1 - \phi } \right)}}$$

where $\phi$ denotes the porosity; $v_{0}$ is the permeation rate (Ergun type); $D_{p}$ is the equivalent grain size, the value of which according to Liu et al.^33^ is taken as *D*_50_ from the grading curve and expressed as the median particle size (Table 2).

**Table 2:** Calculated values of *D*_50_.

*N*	0.1	0.2	0.3	0.4	0.5	0.6	0.7	0.8	0.9
Coefficient value	10.0	5.0	3.3	2.5	2.0	1.7	1.4	1.3	1.1
*D*_50_/mm	0.002	0.063	0.199	0.354	0.500	0.630	0.747	0.841	0.926

The Ergun equation (Ergun 1952) and its extensions are widely used in chemical engineering for flow in porous media. For fluid flow in porous media, the drag coefficient *f* is a function of the Reynolds number Re, e.g., *f* = *F*(Re), the data can be rendered dimensionless and the relationship is expressed as:

10 $$f_{Ergun} = \frac{{150\left( {1 - \phi } \right)}}{{{\text{Re}}_{p} }} + 1.75$$

**Figure 4:**
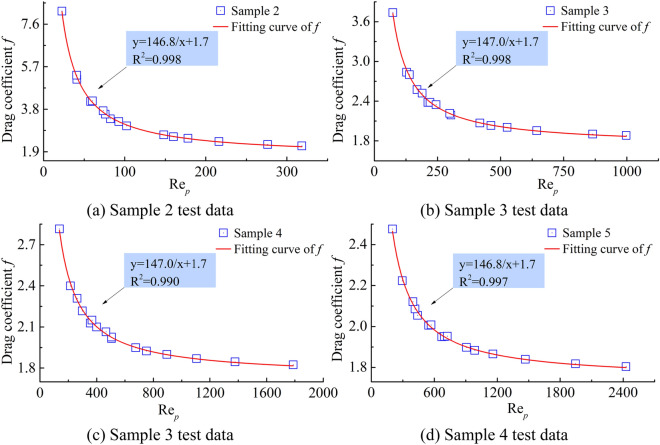
Effect of Reynolds number on drag coefficient.

By inserting the measured data (flow rate and pressure gradient) through the above equations, the parameters of the fluid and the porous medium were derived, including: calculating the drag coefficient and calculating the corresponding Reynolds number. In the coordinates, the curves of Reynolds number *versus* drag coefficient are plotted (Fig. 4).

By calculating the drag coefficient, the graph reflecting the effect of Reynolds number on the drag coefficient was obtained, as shown in Fig. 4. From the analysis of the graph, the resistance coefficients are mainly distributed between 1.0 and 9, the relationship between the drag coefficient and Reynolds number curve can be divided into two stages: in the initial stage, the flow velocity is small and Reynolds number is also small, the drag coefficient decreases with the increase in Reynolds number (following Darcy’s law); in the later stage, the flow velocity increases, Reynolds number also increases, the drag coefficient decreases with the reduction in Reynolds number (denoting a non-Darcian seepage stage). The relationship between them can be fitted by the inverse proportional function, $f = 146.9{/}{\text{Re}}_{p} + 1.7$.

### The distribution of product *βK* during infiltration

In this particular porous medium made of crushed coal grains, the fluid often has to pass through the narrow and tortuous space of the pore throat, where the size of the channels is much smaller than the grain diameter. However, in the Ergun equation, *βK* is about 1.2% of the grain diameter, where the factor *C* is defined here as the shape factor. It is much smaller than the value calculated by Barree et al.^34^.

11 $$\left( {\beta K} \right)_{Ergun} = \frac{{0.012D_{p} }}{{\left( {1 - \phi } \right)}}{ = }CD_{p}$$

The reason for this is that during the percolation of porous media, the fluid flows in the zig-zag space of the pore throat, which is much smaller in width than the grain diameter. When the grain diameter is in the order of millimetres, *βK* is generally distributed within the order of 10^−5^ m. The relationship between *βK*, permeability, and porosity is shown in Eq. (12).

12 $$\left( {\beta K} \right)_{Ergun} = 0.14\sqrt {{K \mathord{\left/ {\vphantom {K {\phi^{3} }}} \right. \kern-\nulldelimiterspace} {\phi^{3} }}}$$

**Figure 5:**
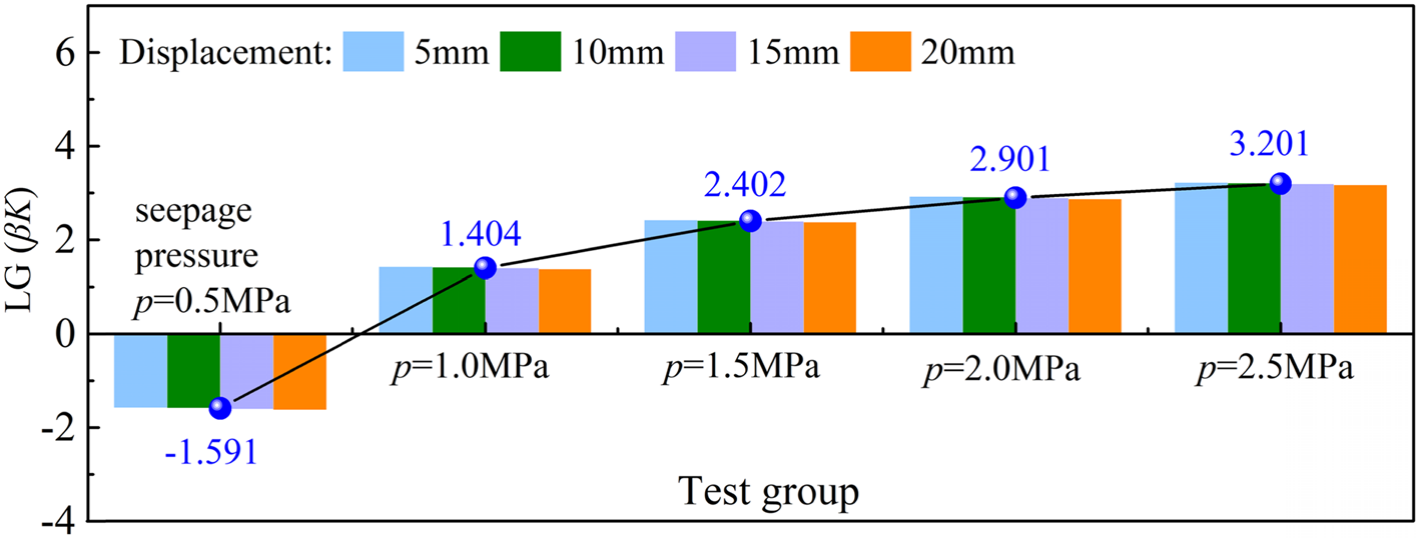
Relationship between *β* and *K* in sample A.

In the analysis, the LG(*βK*) values of the specimens at a downward pressure displacement of 5 mm were selected as the discussion points and coded Alpha 1, Alpha 2, Alpha 3, and Alpha 4. The distribution pattern of the LG(*βK*) values of the specimens at a displacement of 5 mm is shown in Fig. 5.

The relationship between *βK* and displacement was investigated and fitted to obtain the function between *βK* and displacement, and the graph shows the variations in *βK* values of different groups of crushed coal specimens when the penetration pressure is increased from 0.5 to 2.5 MPa. Table 3 lists data pertaining to the distribution of LG(*βK*) values of specimens taken at a displacement of 5 mm.

**Table 3:** Distribution of LG(*βK*) values for specimens at a displacement of 5 mm.

Code	Seepage pressure (MPa)	Value interval	Average value	Fitting function	*R*^2^
Alpha 1	0.5	[− 1.62 to − 1.57]	− 1.591	*y* = − 0.003*x* − 1.551	0.968
Alpha 2	1.0	[1.38–1.43]	1.404	*y* = − 0.003*x* + 1.444	0.968
Alpha 3	1.5	[2.38–2.42]	2.402	*y* = − 0.003*x* + 2.442	0.967
Alpha 4	2.0	[2.87–2.92]	2.901	*y* = − 0.003*x* + 2.942	0.915
Alpha 5	2.5	[3.17–3.22]	3.201	*y* = − 0.003*x* + 3.241	0.961

Direct calculation of LG(*βK*) from experimental data can reduce its variability with a mean value of 1.66, and we obtain a mean value of *βK* of 45.7, which matches the functional trend, *y* = − 0.003*x* + 1.704. Due to the compaction effect the effective pore throat size may be changed, which is also related to the selection of particle diameter. Further fragmentation of the particles leads to a change in the circulation path, and after the fragmentation of the particles, the percentage of the larger particles decreases, and the percentage of the smallest particles increases. The redistribution of the flow path causes changes in its permeation parameters. Therefore, the stress state to which the particles are subjected has a substantial effect on the *βk* value.

### Dependence on *F*o–Re in the seepage process

Usually, the Forchheimer equation, which considers both viscous and inertial collisions, is used to describe this phenomenon. The fluid flow in the crushed coal medium can easily enter a non-Darcy flow state characterised by *F*o > 0.1. For *F*o > 0.1, the non-Darcy flow along the direction of crushing contributes more than 10% to the total pressure drop (as shown in Eq. 13).

13 $$Fo = {{\left( {\frac{dp}{{dL}}} \right)_{non - Darcy} } \mathord{\left/ {\vphantom {{\left( {\frac{dp}{{dL}}} \right)_{non - Darcy} } {\left( {\frac{dp}{{dL}}} \right)}}} \right. \kern-\nulldelimiterspace} {\left( {\frac{dp}{{dL}}} \right)}}_{Darcy} = \frac{{K\beta \rho_{f} v_{0} }}{\mu }$$

Recently, Barree pointed out the limitations of the *β* coefficient and the Forchheimer equation and proposed a new model with a plateau state^35^. Based on *βK* in Eq. (12), the relationship between Fo and particle Reynolds number Re in Eq. (14) was derived.

14 $$Fo = C \cdot {\text{Re}}_{p} = \frac{0.012}{{1 - \phi }} \cdot {\text{Re}}_{p}$$

In Eq. (14), Fo is about 1% of the representative value; this can conclude the discussion about when inertia matters or what is the effective flow pattern for Darcy flow. Special attention is paid to the fact that the distribution of the size of the Re_*p*_ values depends to a large extent on the non-uniformity of the porous medium under consideration.

The effective stress *F*o is a function of the permeability *K*:

15 $$Fo = F\left( {{\text{Re}}_{p} } \right)$$

**Figure 6:**
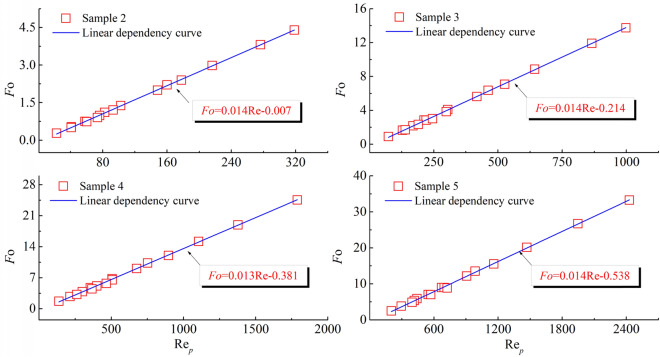
Relationship between Fo and Re for different specimens.

The dependence relationship between the parameters was deduced by further analysing the pattern between the parameters. The relationship between effective stress and permeability was plotted (Fig. 6).

Analysis of the data shows that the relationship between the critical Reynolds number and *F*o can be characterised by using $y = ax + b$. Further, by normalisation, we obtain: $Fo = 0.014{\text{Re}} + 0.285$. It is further shown that the Forchheimer equation and the Ergun equation are essentially the same.

The analysis indicates that different non-linear percolation equations, must all include viscous and inertial effects, and can describe the general flow from low to high velocity in porous media such as laminar, transition, and turbulent flow. The reason is that when the porosity is large, there are more pore channels inside the specimen and the flow velocity is larger, which basically conforms to the linear form of Darcy’s law at this time. When the porosity is small, the pore channels inside the specimen are closed, the flow velocity is small, and the deviation is non-Darcian. When the flow rate is small, the infiltration rate is slow, the Reynolds number is very small, typifying laminar flow, the main source of resistance to fluid flow is the fluid’s own viscosity. When the flow rate gradually increases, the permeability in increased, the Reynolds number gradually increases (albeit slightly), when the laminar flow state no longer exists, the flow trajectory deviates from linear behaviour, the main source of resistance to fluid flow is not only the fluid’s viscosity itself, the effects of inertia on the flow cannot be ignored.

## Conclusion

The permeation parameters of crushed coal grain specimens containing different initial porosities were tested separately by a self-designed lateral limit compression permeation test system, to obtain the variation patterns of several parameters, such as: permeation velocity, Reynolds number, friction factor, *βK*, and Fo; the conclusions are as follows:

1. The calculated Reynolds number can be used as a criterion to judge the non-Darcian flow behaviour. In the test, the Reynolds number distribution of all groups of specimens ranges from 1 to 73. The specimens with porosity in the range of 0.02–0.08 are in the low-permeability zone, showing that 45% of the specimens exhibit Darcy flow in the permeation process. For the specimens with porosity in the range of 0.10–0.14, the flow process is more complex belonging to the conforming flow state.
2. The calculated values of permeability under the definition of the Ergun equation reveal that permeability is an inherent property of a porous medium. At low flow rates, where the pressure drop is proportional to the flow rate and fluid viscosity, the intrinsic permeability was determined according to Darcy’s law. The linear deviation due to inertial loss can be used as a function of porosity to estimate the Reynolds number. The relationship between Reynolds number and drag coefficient was revealed. The results show that the relationship between them could be fitted by an inverse proportional function, *f* = 146.9/Re + 1.7.
3. The determination of the permeability of porous media composed by the crushed coal body can obtain its permeability critical state. In this experiment, the variations in *βK* values of different groups of specimens were ascertained by varying the permeability pressure from 0.5 to 2.5 MPa in a step-by-step manner. Finally, the mean value of *β*K was calculated to be about 45.7, which is consistent with the relationship *y* = − 0.003*x* + 1.704.
4. The dependence between the Forchheimer number Fo and the critical Reynolds number was determined, and the distribution was found to conform to a quasi-linear relationship. Compared with the permeability calculation method used in traditional research, the flow transition process of laminar, transition, and turbulent flow can be possibly described in a direct way at this stage by the variations in these two parameters. That is, the flow characteristics of fluids in porous media from low to high velocity.
